# Safety of beta-blocker and calcium channel blocker antihypertensive drugs in pregnancy: a Mendelian randomization study

**DOI:** 10.1186/s12916-022-02483-1

**Published:** 2022-09-06

**Authors:** Maddalena Ardissino, Eric A. W. Slob, Skanda Rajasundaram, Rohin K. Reddy, Benjamin Woolf, Joanna Girling, Mark R. Johnson, Fu Siong Ng, Dipender Gill

**Affiliations:** 1 National Heart and Lung Institute, Imperial College London, London, UK; 2 Nuffield Department of Population Health, University of Oxford, Oxford, UK; 3 Medical Research Council Biostatistics Unit, University of Cambridge, Cambridge, UK; 4 Department of Applied Economics, Erasmus School of Economics, Erasmus University Rotterdam, Rotterdam, the Netherlands; 5 Erasmus University Rotterdam Institute for Behavior and Biology, Erasmus University Rotterdam, Rotterdam, the Netherlands; 6 Centre for Evidence-Based Medicine, University of Oxford, Oxford, UK; 7 Faculty of Medicine, Imperial College London, London, UK; 8 School of Psychological Science, University of Bristol, Bristol, UK; 9 Medical Research Council Integrative Epidemiology Unit, University of Bristol, Bristol, UK; 10 Faculty of Epidemiology and Population Health, London School of Hygiene and Tropical Medicine, London, UK; 11 Chelsea and Westminster Hospital NHS Foundation Trust, West Middlesex Hospital, London, UK; 12 Division of Reproductive and Developmental Biology, Department of Metabolism, Digestion and Reproduction, Imperial College London, London, UK; 13 Department of Epidemiology and Biostatistics, School of Public Health, Medical School Building, St Mary’s Hospital, Imperial College London, W2 1PG London, UK; 14 Chief Scientific Office, Research and Early Development, Novo Nordisk, Copenhagen, Denmark

**Keywords:** Blood pressure, Beta-blocker, Calcium channel blocker, Mendelian randomization, Pregnancy

## Abstract

**Background:**

Beta-blocker (BB) and calcium channel blocker (CCB) antihypertensive drugs are commonly used in pregnancy. However, data on their relative impact on maternal and foetal outcomes are limited. We leveraged genetic variants mimicking BB and CCB antihypertensive drugs to investigate their effects on risk of pre-eclampsia, gestational diabetes and birthweight using the Mendelian randomization paradigm.

**Methods:**

Genetic association estimates for systolic blood pressure (SBP) were extracted from summary data of a genome-wide association study (GWAS) on 757,601 participants. Uncorrelated single-nucleotide polymorphisms (SNPs) associated with SBP (*p* < 5 × 10^−8^) in BB and CCB drug target gene regions were selected as proxies for drug target perturbation. Genetic association estimates for the outcomes were extracted from GWASs on 4743 cases and 136,325 controls (women without a hypertensive disorder in pregnancy) for pre-eclampsia or eclampsia, 7676 cases and 130,424 controls (women without any pregnancy-related morbidity) for gestational diabetes, and 155,202 women (who have given birth at least once) for birthweight of the first child. All studies were in European ancestry populations. Mendelian randomization estimates were generated using the two-sample inverse-variance weighted model.

**Results:**

Although not reaching the conventional threshold for statistical significance, genetically-proxied BB was associated with reduced risk of pre-eclampsia (OR per 10 mmHg SBP reduction 0.27, 95%CI 0.06–1.19, *p* = 0.08) and increased risk of gestational diabetes (OR per 10 mmHg SBP reduction 2.01, 95%CI 0.91–4.42, *p* = 0.08), and significantly associated with lower birthweight of first child (beta per 10 mmHg SBP reduction − 0.27, 95%CI − 0.39 to − 0.15, *p* = 1.90 × 10^−5^). Genetically-proxied CCB was associated with reduced risk of pre-eclampsia and eclampsia (OR 0.62, 95%CI 0.43–0.89, *p* = 9.33 × 10^−3^), and was not associated with gestational diabetes (OR 1.05, 95% CI 0.76–1.45, *p* = 0.76) or changes in birthweight of first child (beta per 10 mmHg SBP reduction 0.02, 95%CI − 0.04–0.07, *p* = 0.54).

**Conclusions:**

While BB and CCB antihypertensive drugs may both be efficacious for lowering blood pressure in pregnancy, this genetic evidence suggests that BB use may lower birthweight. Conversely, CCB use may reduce risk of pre-eclampsia and eclampsia without impacting gestational diabetes risk or birthweight. These data support further study on the effects of BBs on birthweight.

**Supplementary Information:**

The online version contains supplementary material available at 10.1186/s12916-022-02483-1.

## Background

Hypertensive disorders of pregnancy are a major cause of maternal and foetal morbidity [1–3]. Recent randomised clinical trial (RCT) data provide evidence that treatment of mild chronic hypertension in pregnancy reduces maternal morbidity without impacting birthweight [4]. However, multiple past observational studies have described an association between the use of individual antihypertensive drugs during pregnancy and low birthweight [5–7].

Despite guideline-directed use of the beta-blocker (BB), labetalol, and the calcium channel blocker (CCB), nifedipine, for use as antihypertensive drugs in pregnancy [8], there is little evidence regarding their comparative efficacy [9] and safety [10]. The most recent Cochrane review highlighted that RCT evidence is currently insufficient to reliably estimate the comparative benefits and adverse effects of these antihypertensive drugs [10].

In the setting of limited clinical trial evidence, the Mendelian randomization (MR) paradigm may be used to provide insight into the effects of these pharmacological interventions [11]. The MR framework uses genetic variants associated with exposures as instrumental variables to study causal relationships between exposures and outcomes. MR facilitates stronger causal inference compared with conventional observational analyses, as inheritance of genetic variants predisposing to an exposure occurs randomly at conception, similar to randomization of an intervention in a clinical trial. This paradigm can be extended to explore the effects of drug target perturbation by leveraging the natural sequence variation in genes encoding drug targets [11]. This can offer insight into target-based efficacy and adverse effects.

In this study, we identified and leveraged genetic variants that mimic BB and CCB antihypertensive drugs to investigate their effects on risk of pre-eclampsia or eclampsia, gestational diabetes and birthweight.

## Results

All genetic variants used as instrumental variables had F-statistics above 10 (Table 1). The genetic variants used as instruments for SBP showed a strong correlation in their associations for SBP and DBP (Additional file 1: Table S1).

**Table 1:** Gene regions and corresponding positions used for different drug targets

Exposure drug target	Gene	Gene position (GRCh37/hg19)	#SNPs (*p* < 5 × 10^−8^, *r*^2^ < 0.1)	*R*^2^	F-statistic
Beta_1_-adrenoceptor blockers	ADRB1	Chr10: 115,803,625–115,806,663	2	0.00028	105.49
Calcium channel blockers	CACNA1C	Chr3: 53,528,638–53,847,760	4	0.00029	55.09
	CACNA1D	ChrX: 49,061,523–49,089,802	0	-	-
	CACNA1F	Chr7: 81,575,760–82,073,272	0	-	-
	CACNA1S	Chr3: 50,400,044–50,541,675	0	-	-
	CACNA2D1	Chr1: 201,008,640–201,081,554	0	-	-
	CACNA2D2	Chr17: 37,329,706–37,353,922	0	-	-
	CACNB1	Chr10: 18,429,353–18,832,486	16	0.0015	72.81
	CACNB2	Chr12: 49,208,263–49,222,724	1	9.28e × 10^−05^	70.28
	CACNB3	Chr2: 152,689,285–152,955,681	0	-	-
	CACNB4	Chr17: 65,040,670–65,052,913	0	-	-
	CACNG1	Chr12: 2,162,153–2,807,116	2	0.0020	38.69

F-statistic for systolic blood pressure is 62.77. Gene position obtained using DBSNP. Reported windows do not include ± 10kB window. F-statistic calculated using the formula $F=\frac{(n-k-1)}{k} \frac{({R}^{2})}{(1-{R}^{2})}$, where we calculated ${R}^{2}$ by summing SNP-wise ${R}^{2}$ (using the formula ${R}^{2}= \frac{F}{(N-2+F)}$, with $F={\left(\frac{\beta }{SE(\beta )}\right)}^{2}$) to find joint ${R}^{2}$

*Abbreviations*: *chr* chromosome, *“-”* not available

### Systolic blood pressure

**Fig. 1:**
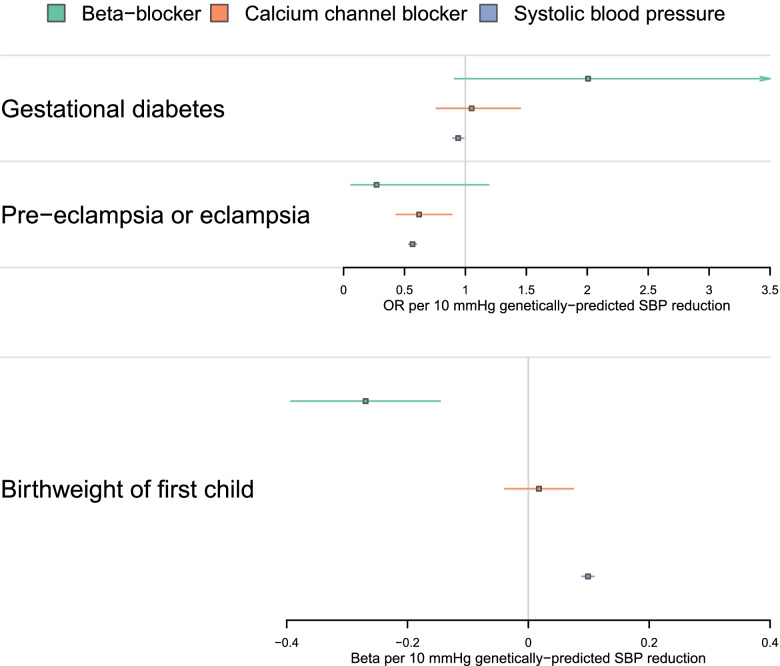
Mendelian randomization estimates (scaled to 10-mmHg systolic blood pressure reduction) for beta-blocker and calcium channel blocker drug effects, and systolic blood pressure reduction by any mechanism

Genetically-proxied reduction in SBP by 10 mmHg through any mechanism was associated with lower risk of pre-eclampsia and eclampsia (OR per 10 mmHg reduction in SBP 0.57, 95%CI 0.53–0.60, *p* = 1.11 × 10^−84^), lower risk of gestational diabetes (OR per 10 mmHg reduction in SBP 0.94, 95%CI 0.90–0.99, *p* = 0.01), and an increase in birthweight category (foetal birthweight increased up by 0.10 categories on average per 10 mmHg reduction in SBP; beta 0.10, 95%CI 0.09–0.11, *p* = 9.54 × 10^−80^). The results are shown in Fig. 1 and Additional file 1: Table S2.

### Beta-blocker

**Fig. 2:**
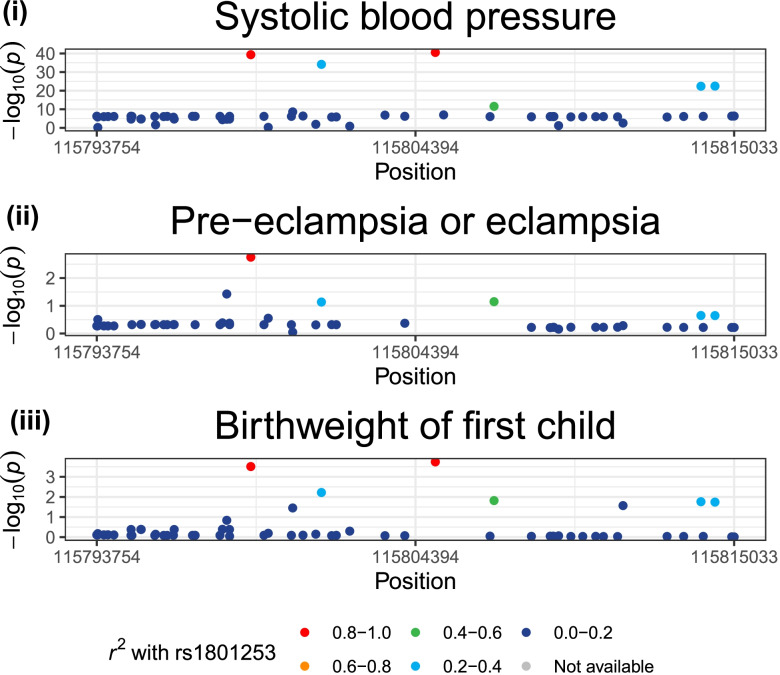
Locus plots to illustrate evidence of genetic colocalization between systolic blood pressure and pregnancy outcomes, showing GWAS $-{\mathrm{log}}_{10}p$ values at the *ADRB1* locus on (i) systolic blood pressure, (ii) pre-eclampsia or eclampsia, and (iii) birthweight of the first child

Genetically-proxied BB effect was associated with reduced risk of pre-eclampsia and eclampsia (OR per 10 mmHg reduction in SBP 0.27, 95%CI 0.06–1.19, *p* = 0.08), although this did not reach the conventional threshold to qualify as statistically significant (Fig. 1 and Additional file 1: Table S2). Colocalization analysis supported a shared causal variant for the two traits (posterior probability for H4: 60.3%; Additional file 1: Table S3). There was also an association with a higher risk of gestational diabetes (OR per 10 mmHg reduction in SBP 2.01, 95%CI 0.91–4.42, *p* = 0.08), although this does not reach a conventional threshold to qualify as statistically significant and the colocalization analysis did not provide evidence for a shared causal variant (posterior probability H3: 0.14%, posterior probability H4: 3.33%). Genetically-proxied BB effect was associated with lower birthweight category (foetal birthweight reduced by 0.27 categories on average per 10 mmHg SBP reduction; beta − 0.27, 95%CI − 0.39 to − 0.15, *p* = 1.90 × 10^−5^) and colocalization analysis supported a shared causal variant for the two traits (posterior probability for H4: 69.3%). A locus plot for *ADRB1* with systolic blood pressure, pre-eclampsia and eclampsia, and birthweight is provided in Fig. 2.

### Calcium channel blocker

Genetically-proxied CCB effect was associated with lower risk of pre-eclampsia or eclampsia (OR per 10 mmHg reduction in SBP 0.62, 95%CI 0.43–0.89, *p* = 9.33 × 10^−3^), but was not associated with gestational diabetes (OR 1.05, 95%CI 0.76–1.45, *p* = 0.76) or birthweight category (0.02 higher category per 10 mmHg reduction in SBP, 95%CI − 0.04–0.07, *p* = 0.54). Full results are shown in Fig. 1, Table 2, and Additional file 1: Tables S2 and S3.

**Table 2:** Inverse-variance weighted two-sample Mendelian randomization estimates for antihypertensive drug effects and systolic blood pressure reduction through any mechanism (scaled to 10 mmHg) on the set of outcomes

**Exposure/drug target**	**Outcome**	**OR**^a^	**LCI 95%**	**UCI 95%**	***P*-value**
Beta-blocker mediated 10 mmHg SBP reduction (# SNPs = 2)	Pre-eclampsia or eclampsia	0.27	0.06	1.19	0.08
	Gestational diabetes	2.01	0.91	4.42	0.08
	Birthweight^a^	− 0.27	− 0.39	− 0.15	1.90 × 10^−05^
Calcium channel blocker mediated 10 mmHg SBP reduction (# SNPs = 23)	Pre-eclampsia or eclampsia	0.62	0.43	0.89	9.33 × 10^−3^
	Gestational diabetes	1.05	0.76	1.45	0.76
	Birthweight^a^	0.02	− 0.04	0.07	0.54
Systolic blood pressure, any mechanism (10 mmHg reduction, # SNPs = 1336–1346)	Pre-eclampsia or eclampsia	0.57	0.53	0.60	1.11 × 10^−84^
	Gestational diabetes	0.94	0.90	0.99	0.01
	Birthweight^a^	0.10	0.09	0.11	9.54 × 10^−80^

*Abbreviations*: *OR* Odds ratio, *LCI* Lower bound for 95% confidence interval, *UCI* Upper bound for 95% confidence interval

^a^beta coefficient reported for birthweight

### Sensitivity analyses

Sensitivity analyses using SBP genetic association estimates from females only (Additional file 1: Tables S4 and S5) and DBP genetic association estimates from females only (Additional file 1: Tables S6 and S7) produced similar results. Our findings were also robust to the choice of joint prior in colocalization analysis (Additional file 1: Table S8). Finally, MR methods more robust to the violation of requisite assumptions also produced similar results (Additional file 1: Tables S9-S11). Further details are provided in Additional file 2: Supplementary Note.

## Discussion

We leveraged genetic variants mimicking BB and CCB drug classes to investigate their respective effects on pre-eclampsia or eclampsia, gestational diabetes and birthweight. Our results support the assertion that BB antihypertensive drugs may lower birthweight, by providing consistent results in MR analysis and evidence of a shared causal variant in colocalization analysis. Caution and pharmacovigilance may therefore be warranted regarding use of BB antihypertensive drugs in pregnancy. Conversely, our genetic evidence supported that CCB reduced risk of pre-eclampsia or eclampsia, but had no apparent impact on birthweight category or gestational diabetes. CCBs may therefore be the preferred first-line agent to treat hypertension in pregnancy, because of a potentially superior safety profile (Table 2).

Treatment of mild hypertension in pregnancy has been shown to lead to lower risk of adverse maternal outcomes in a recent RCT [4]. In this study, the majority of patients were given labetalol or nifedipine as the first-line antihypertensive agent. These antihypertensive drugs, BB and CCB agents respectively, are recommended by the National Institute for Health and Care Excellence (NICE) in the UK as first- and second-line agents for hypertension in pregnancy [8]. However, evidence regarding their comparative safety and efficacy is scarce [10]. Historically, ethical concerns surrounding RCTs in pregnancy have precluded their implementation, especially in the setting of primary prevention [12]. Where RCTs have been undertaken, they often lack sufficient enrolment to detect adverse effects. For the outcome of birthweight, a trial sequential analysis suggested that 7030 participants would be required to run an adequately powered trial comparing labetalol to nifedipine [9]. Such recruitment has not previously been reached in RCTs in this field. Considering the increasing prevalence of hypertension in pregnancy [2] and the severity of the related adverse outcomes, the lack of evidence represents a major obstacle towards optimising clinical practice.

Given the current absence of robust RCT data in pregnancy, large-scale observational data have been used to inform clinical practice. In confounder-adjusted analyses of almost 380,000 singleton pregnancies from an ethnically diverse American cohort, use of labetalol was associated with an increased likelihood of a small for gestational age birth [5]. Our results are consistent with this finding, thus highlighting the importance of ongoing pharmacovigilance. Furthermore, recent individual participant meta-analysis data outside of pregnancy show that over 4.5 years, BB use increases the likelihood of developing type 2 diabetes while CCB use does not [13]. This propensity of BB use to increase diabetes liability was also supported in our current study. Given that genetically-proxied CCB use was not associated with either reduced birthweight category or an increased risk of gestational diabetes, our findings suggest that CCBs may be preferred to BBs in the management of hypertension in pregnancy.

A limitation of leveraging genetic variants to investigate drug effects in pregnancy is that they exert small lifelong effects, whereas pharmacological interventions typically exert effects of larger magnitude but over a discrete period of the life course. For this reason, MR analysis should only be used to identify potential clinical effects, but not to infer their magnitude. Furthermore, our analysis may have been limited by low statistical power, which can be gauged by the confidence intervals of our MR estimates. There is also the theoretical possibility that genetic variants used to proxy the exposures may also be exerting pleiotropic effects through the off-spring, thus biassing the MR results. In addition, as this study was performed using data from European ancestry populations, the results may not be generalizable to populations of other ancestral backgrounds. Our main SBP GWAS considered both male and female participants, whereas the outcome datasets are in females. To address this, we repeated our analysis using SBP GWAS in females only. Findings were similar when we restricted our exposure dataset to female participants. This female-only GWAS did not adjust for body mass index or correct for antihypertensive medication use (as our main SBP GWAS did), so these findings also demonstrate robustness against the choice of GWAS covariates. Finally, the considered outcome of birthweight was not adjusted by gestational age at birth. This means that the ‘low birthweight’ category may include both infants who are small for-gestational age or born preterm. Nevertheless, these are both important outcomes of interest as they are known foetal adverse effects of maternal hypertension in pregnancy.

In summary, using large-scale genetic association data, we find evidence supporting that maternal BB antihypertensive drug use during pregnancy may lower birthweight. Our findings provide an important rationale for pharmacovigilance studies on BBs, and RCTs comparing BB and CCB drugs in pregnancy.

## Methods

### Data sources

**Fig. 3:**
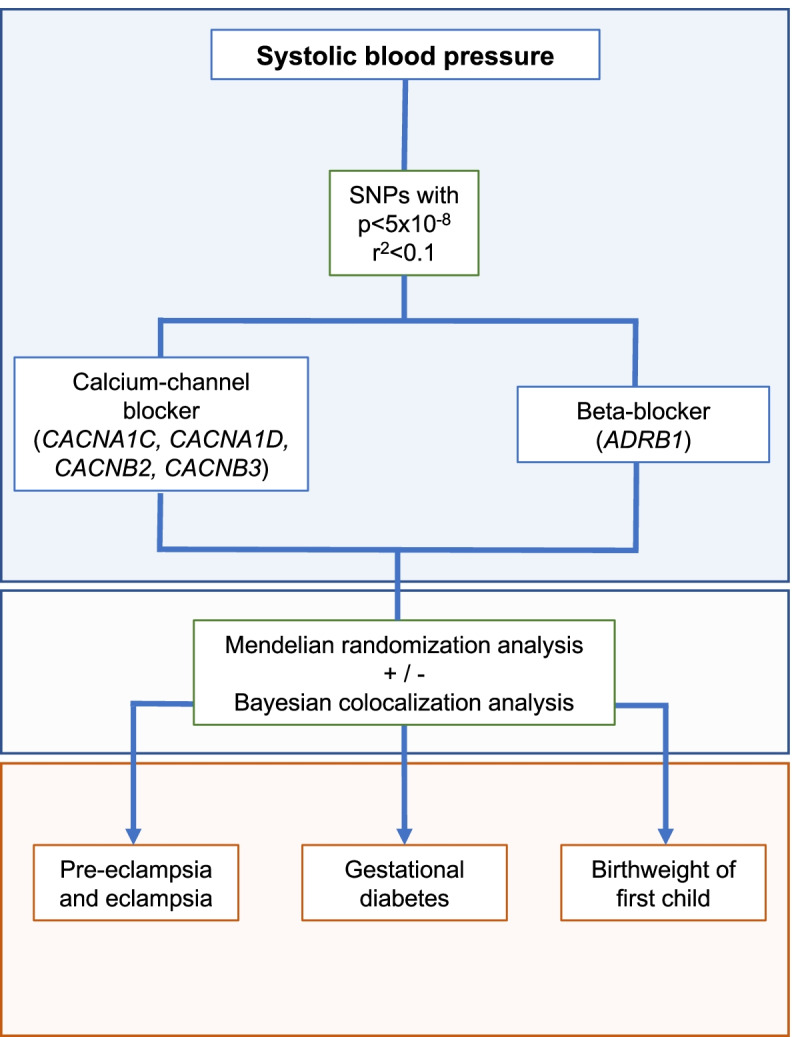
Study design flowchart

A flowchart depicting the statistical analysis plan is displayed in Fig. 3. Publicly available genetic association summary data were used for all analyses. Appropriate ethical approval and participant consent were obtained in the original studies that generated the data. A summary of the data sources used in this study is presented in Table 3. Genetic association estimates were extracted from summary data of GWAS studies on 757,601 participants for systolic blood pressure (SBP; corrected for blood pressure-lowering medication use and body mass index) [14], 4743 pre-eclampsia or eclampsia cases (identified using registry data on International Classification of Diseases (ICD) 8,9, and 10 codes) and 136,325 controls (women without a hypertensive disorder in pregnancy) [15], 7676 gestational diabetes cases (identified using registry data on ICD codes 9 and 10) and 130,424 controls (women without any pregnancy-related morbidity) [15], and 155,202 women who have given birth at least once for birthweight of the first child [16]. The birthweight of the first child variable was split into three bins, which are coded in 3 categories as follows: 0: birthweight of first child below 7 pounds, 1: birthweight of the first child is 7 pounds, 2: birthweight of the first child is above 7 pounds. All studies were performed in European ancestry populations. All GWASs were corrected for age, sex (where applicable) and principal components.

**Table 3:** Information on the studies and consortia from which genetic association data were obtained

Exposure	Phenotype name in study	Study	Ancestry	*N* cases (/controls)	Controls definition/selection criteria	Unit	PMID/link
**Exposure data**							
Systolic blood pressure	Systolic blood pressure	Evangelou et al., 2018	European	757,601	-	mmHg	30,224,653
**Outcome data**							
Pre-eclampsia or eclampsia	Pre-eclampsia or eclampsia	FINNGEN DF6	European	4743/136,325	Women without a hypertensive disorder in pregnancy	Log(OR) (yes vs no)	https://finngen.gitbook.io/documentation
Gestational diabetes	Diabetes mellitus in pregnancy	FINNGEN DF6	European	7676/130,424	Women without any pregnancy-related morbidity	Log(OR) (yes vs no)	https://finngen.gitbook.io/documentation
Birthweight of first child	First child birthweight	Neale Lab	European	155,202	Women who indicated they had given birth to at least one child (based on UKB data-field 2744)	Birthweight in pounds, split into three ordered categorical variable bins: • 0 for < 7 pounds • 1 for 7 pounds • 2 for > 7 pounds	http://www.nealelab.is/uk-biobank/

### Instrumental variables

For each drug target, single-nucleotide polymorphisms (SNPs) in the drug target gene region (± 10kB) that were associated with SBP (p < 5 × 10^−8^) were selected as proxies for drug target perturbation after clumping to a pairwise linkage disequilibrium threshold of *r*^2^ < 0.1 using the 1000 Genomes European reference panel. For SNPs reflecting SBP reduction by any mechanism, we took all genome-wide significant hits from the GWAS. These variants were also clumped a to a pairwise linkage disequilibrium threshold of *r*^2^ < 0.1 using the 1000 Genomes European reference panel, to mimic the approach taken for the drug targets as closely as possible. A flowchart showing the number of SNPs at each stage is shown in Additional file 2: Fig. S1. The full list of SNPs for the different targets are presented in Additional file 1: Tables S1, and S12-S17. The gene locations for BB and CCB drug targets are reported in Table 1.

### Mendelian randomization

MR estimates for SBP reduction through the two classes of antihypertensive medications on pregnancy outcomes were generated using the two-sample inverse-variance weighted (IVW) model [17] with standard error correction for under dispersion. This was implemented in the *TwoSampleMR* package version 0.5.6 [18], in R version 4.1.0 [19]. If there was only a single variant available, we used the Wald ratio to estimate the effect. The results are reported as odds ratios (OR) or beta coefficients scaled per 10 mmHg SBP reduction with 95% confidence intervals (95%CI). MR is a method that, under three core assumptions, intends to estimate causal effects. These three core assumptions are: each variant must be associated with the exposure (relevance assumption), each variant is not associated with any potential confounding variable (independence assumption), and each variant must be only associated with the outcome via the risk factor (exclusion restriction) [20]. An F-statistic value above 10 is used as a rule of thumb in the instrumental variables literature to support that the relevance assumption seems to be met. For the two other assumptions (independence assumption and exclusion restriction), it is somewhat more difficult to demonstrate their validity. Since we study known drug targets for lowering SBP, this improves our confidence in using variants associated with SBP in the genes coding for these drug targets. Additionally, we implement methods more robust to violations of the conventional Mendelian randomization assumptions: MR-Egger analysis, the weighted median method and MR-PRESSO. The methods are described in the Additional file 2: Supplementary Note.

Since 3 different outcomes (pre-eclampsia or eclampsia, gestational diabetes, and birthweight category of the first child) were tested, a Bonferroni correction was applied when interpreting the *p*-value for statistical significance 0.05/3 = 0.017.

### Colocalization

For the BB gene region (*ADRB1*), a Bayesian test for genetic colocalization was also performed to investigate the likelihood that the exposure-outcome pair had a shared versus distinct causal variants [21]. For these analyses, the R package *coloc* (version 5.1.0) was used [21]. Approximate Bayes Factor Colocalization analyses (using the coloc.abf function) were carried out, which assumes that there is at most 1 causal SNP in the gene region for each trait. The method allows for the following hypotheses:

- H0: no causal variants in the gene region.
- H1: only trait 1 has a causal variant in the gene region.
- H2: only trait 2 has a causal variant in the gene region.
- H3: both traits have a different causal variant in the gene region.
- H4: both traits have a shared causal variant in the gene region.

Default priors were utilised as prior probabilities of the different hypotheses: ${p}_{1}={p}_{2}={10}^{-4}$,${p}_{12}={10}^{-5}$. Using the data and priors, the method calculates the posterior probabilities for the different hypotheses [21]. In Additional file 2: Supplementary Note, we discuss the use of priors in more depth and rerun our analyses using different values for the joint prior ${p}_{12}$.

Evidence for a shared causal variant was concluded when the posterior probability for H4 surpasses a value of 0.5 (this means that a posteriori a shared causal variant is more likely than distinct causal variants). If a shared causal variant was found for the pair, this contributes further evidence that the antihypertensive drug target and outcome share a causal mechanism, and that any identified MR association is unlikely to be attributable to genetic confounding through a variant in linkage disequilibrium. This analysis was not performed for the CCB target as a whole, because this is made up of several proteins that are coded for by different genes. However, we do inspect each CCB region separately for completeness.

Given that colocalization is being performed to explore the robustness of an MR association to possible genetic confounding, a H4 > 0.5 was considered to represent evidence of colocalization, as this would suggest that there is more evidence of a shared causal variant than distinct causal variants underlying the MR association between the exposure and the outcome.

### Sensitivity analyses

To additionally explore the robustness of our findings to consideration of blood pressure genetic association estimates specific to females, or related to diastolic blood pressure (DBP) rather than SBP, we additionally repeated the analyses using instruments selected from GWAS data obtained in females, and on DBP rather than SBP. Full details are provided in the Additional file 2: Supplementary Note.

## Supplementary Information

**Additional file 1: Table S1.** Instrumental variables for systolic blood pressure. **Table S2.** Mendelian randomization results. **Table S3.** Colocalization results. **Table S4.** Mendelian randomization results in analyses considering females only. **Table S5.** Colocalization results in analyses considering females only. **Table S6.** Mendelian randomization results in analyses considering diastolic blood pressure in females only. **Table S7.** Colocalization results in analyses considering diastolic blood pressure in females only. **Table S8.** Colocalization results considering alternative priors. **Table S9.** MR-Egger results. **Table S10.** Weighted median results. **Table S11.** MR-PRESSO results. **Table S12.** Instrumental variables selected at the ADRB1 locus. **Table S13.** Instrumental variables selected at the CACNA1C locus. **Table S14.** Instrumental variables selected at the CACNA1D locus. **Table S15.** Instrumental variables selected at the CACNB2 locus. **Table S16.** Instrumental variables selected at the CACNB3 locus. **Table S17.** Instrumental variables selected at all calcium channel blocker gene regions.

**Additional file 2: **Supplementary Note: Additional details on the methodology. **Figure S1.** Flow chart detailing instrumental variable selection.

## Data Availability

The study protocol was not pre-registered. The datasets supporting the conclusions of this article are publicly available. Summary statistics for the systolic blood pressure genome-wide association study (GWAS) are available from https://grasp.nhlbi.nih.gov/downloads/ResultsAug2019/2018/Evangelou/UKB-ICBPmeta750k_SBPsummaryResults.txt.gz with corresponding readme file on https://grasp.nhlbi.nih.gov/downloads/ResultsAug2019/2018/Evangelou/Evangelou_README_BPGWAS.txt. Summary statistics for the birthweight of the first child trait GWAS using UK Biobank data are available from https://broad-ukb-sumstats-us-east-1.s3.amazonaws.com/round2/additive-tsvs/2744.gwas.imputed_v3.female.tsv.bgz, for systolic blood pressure on https://broad-ukb-sumstats-us-east-1.s3.amazonaws.com/round2/additive-tsvs/4079_raw.gwas.imputed_v3.female.tsv.bgz, and for diastolic blood pressure on https://broad-ukb-sumstats-us-east-1.s3.amazonaws.com/round2/additive-tsvs/4080_raw.gwas.imputed_v3.female.tsv.bgz. The documentation for these data sources can be found in https://broad-ukb-sumstats-us-east-1.s3.amazonaws.com/round2/additive-tsvs/README. Summary statistics for pre-eclampsia or eclampsia GWAS are available from FinnGen (https://storage.googleapis.com/finngen-public-data-r6/summary_stats/finngen_R6_O15_PRE_OR_ECLAMPSIA.gz) and similarly from gestational diabetes (https://storage.googleapis.com/finngen-public-data-r6/summary_stats/finngen_R6_O15_PREG_DM.gz). Detailed documentation is provided on https://www.finngen.fi/en/access_results. Statistical code is available from the corresponding author on reasonable request.

## References

[1] Garovic VD, White WM, Vaughan L, Saiki M, Parashuram S, Garcia-Valencia O (2020). Incidence and long-term outcomes of hypertensive disorders of pregnancy. J Am Coll Cardiol.

[2] Corrigan L, O’Farrell A, Moran P, Daly D (2021). Hypertension in pregnancy: Prevalence, risk factors and outcomes for women birthing in Ireland. Pregnancy Hypertens.

[3] Xiong X (2002). Impact of preeclampsia and gestational hypertension on birth weight by gestational age. Am J Epidemiol.

[4] Tita AT, Szychowski JM, Boggess K, Dugoff L, Sibai B, Lawrence K, et al. Treatment for mild chronic hypertension during pregnancy. N Engl J Med. 2022. 10.1056/NEJMoa2201295. 10.1056/NEJMoa2201295 PMC9575330 35363951

[5] Duan L, Ng A, Chen W, Spencer HT, Lee M-S (2018). Beta-blocker subtypes and risk of low birth weight in newborns. J Clin Hypertens.

[6] Magee LA, von Dadelszen P, Rey E, Ross S, Asztalos E, Murphy KE (2015). Less-tight versus tight control of hypertension in pregnancy. N Engl J Med.

[7] von Dadelszen P, Ornstein M, Bull S, Logan A, Koren G, Magee L (2000). Fall in mean arterial pressure and fetal growth restriction in pregnancy hypertension: a meta-analysis. Lancet.

[8] NICE 2019. Hypertension in pregnancy: diagnosis and management [NG 133]. National Institute for Health and Care Excellence (NICE); https://www.nice.org.uk/guidance/ng133. 2019; https://www.nice.org.uk/guidance/ng133. http://www.nice.org.uk/guidance/cg107%5Cn;https://www.dovepress.com/getfile.php?fileID=7818%5Cn; http://www.ijgo.org/article/S0020-7292(02)80002-9/abstract. Accessed 10 Mar 2022. Last Accessed on 2022–03–10.

[9] Bone JN, Sandhu A, Abalos ED, Khalil A, Singer J, Prasad S (2022). Oral antihypertensives for nonsevere pregnancy hypertension: systematic review, network meta- and trial sequential analyses. Hypertension.

[10] Abalos E, Duley L, Steyn DW, Gialdini C (2018). Antihypertensive drug therapy for mild to moderate hypertension during pregnancy. Cochrane database Syst Rev.

[11] Gill D, Georgakis MK, Walker VM, Schmidt AF, Gkatzionis A, Freitag DF (2021). Mendelian randomization for studying the effects of perturbing drug targets. Wellcome Open Res.

[12] Committee on Ethics (2015). Committee opinion no. 646 summary: ethical considerations for including women as research participants. Obstet Gynecol.

[13] Nazarzadeh M, Bidel Z, Canoy D, Copland E, Wamil M, Majert J (2021). Blood pressure lowering and risk of new-onset type 2 diabetes: an individual participant data meta-analysis. Lancet.

[14] Evangelou E, Warren HR, Mosen-Ansorena D, Mifsud B, Pazoki R, Gao H, et al. Genetic analysis of over 1 million people identifies 535 new loci associated with blood pressure traits. Nat Genet. 2018. 10.1038/s41588-018-0205-x. 10.1038/s41588-018-0205-x PMC6284793 30224653

[15] FinnGen. FinnGen consortium, R6 Available https//www.finngen.fi/en/access_results Accessed 04 April 2022.

[16] Neale lab: UK Biobank GWAS Results. Neale lab, available http//www.nealelab.is/uk-biobank Accessed 04 April 2022.

[17] Burgess S, Bowden J, Fall T, Ingelsson E, Thompson SG (2017). Sensitivity analyses for robust causal inference from mendelian randomization analyses with multiple genetic variants. Epidemiology.

[18] Hemani G, Zheng J, Elsworth B, Wade KH, Haberland V, Baird D (2018). The MR-Base platform supports systematic causal inference across the human phenome. Elife.

[19] R Core Team. 2022. R: A language and environment for statistical computing. R Found Stat Comput Vienna, Austria. https://www.R-project.org/.

[20] Bowden J, Del Greco MF, Minelli C, Davey Smith G, Sheehan N, Thompson J (2017). A framework for the investigation of pleiotropy in two-sample summary data Mendelian randomization. Stat Med.

[21] Giambartolomei C, Vukcevic D, Schadt EE, Franke L, Hingorani AD, Wallace C (2014). Bayesian test for colocalisation between pairs of genetic association studies using summary statistics. PLoS Genet.

