# Supplementary material for: Safety of beta-blocker and calcium channel blocker antihypertensive drugs in pregnancy: a Mendelian randomization study
Source: BMC Med. 2022 Sep 6;20:288. doi: 10.1186/s12916-022-02483-1 (PMC9446737; doi:10.1186/s12916-022-02483-1)
Supplement: Supplementary file 2 — Additional file 2: Supplementary Note: Additional details on the methodology. Figure S1. Flow chart detailing instrumental variable selection. [file 12916_2022_2483_MOESM2_ESM.docx]

## Supplementary Note

### **Supplementary analysis to check robustness with respect to study participants in systolic blood pressure GWAS**

When selecting the genetic instruments for BBs and CCBs, given the outcomes are only limited to women, it can be more suitable to select genetic instruments for these drugs using a GWAS only in female participants (N=182,645). Hence, we perform a sensitivity analysis using the Neale lab GWAS in UK Biobank using females only, using data field 4080, which is the automated reading of systolic blood pressure (SBP). This GWAS is corrected for the first 10 principal components. Results are presented in **Additional file 1: Tables S4 and S5**. Since this GWAS has fewer participants, we have a lower number of significant single-nucleotide polymorphisms (SNPs) (and hence lower statistical power). For CACNA1C and CACNB3 we have no significant SNPs, so we could not do a Mendelian randomization analysis for these targets.

The results for genetically-proxied reduction of SBP by any mechanism is associated with pre-eclampsia and eclampsia (OR per 10mmHg reduction in SBP 0.58, 95%CI 0.52-0.65, p=4.67x10^-22^), and birthweight of the first child (beta per 10 mmHg reduction 0.11, 95%CI 0.09-0.14, p=6.20x10^-27^). These results are very similar to before. The association with gestational diabetes is no longer significant (OR per 10mmHg reduction in SBP 0.96, 95%CI 0.88-1.04, p=0.365). This could be due to less precision as the sample size is smaller (now N=182,647, N=757,601 in the main analysis).

For ADRB1, we observe that genetically-proxied reduction of SBP has a significant association with both pre-eclampsia and eclampsia (OR per 10mmHg reduction in SBP 0.13, 95%CI 0.03-0.48, p=0.002), and birthweight of the first child (beta per 10 mmHg reduction -0.306, 95%CI -0.497 - -0.145, p=1.81x10^-4^). For gestational diabetes, no significant effect is observed (OR per 10mmHg reduction in SBP 2.30, 95%CI 0.81-6.54, p=0.118). For the colocalization analyses, we observe that ADRB1 still colocalizes with both pre-eclampsia and eclampsia (posterior probability H4: 59.40%) and birthweight of first child (posterior probability H4: 64.93%).

For CACNA1D, we observe that the results are qualitatively similar as before, however in this case there seems to be some evidence for a significant association with birthweight of the first child, but this significance disappears after correcting for multiple testing(beta per 10 mmHg reduction 0.203, 95%CI 0.038 – 0.368, p=0.016). For CACNB2, there is again a significant association with pre-eclampsia and eclampsia. For calcium channel blockers in general, the association with pre-eclampsia and eclampsia does not reach conventional levels of significance (p=0.324), which it did reach in the main analysis. In the calcium channel blocker gene regions, there is no strong evidence for colocalization, which aligns with the previous results.

### **Supplementary analysis to check robustness with respect to diastolic blood pressure**

Since we use antihypertensive targets in our analysis that work not only through lowering SBP but also by lowering diastolic blood pressure (DBP), we reran our analysis where we instead use DBP to measure our exposure. We repeated our analysis using the DBP GWAS (N=182,647) from Neale lab in UK Biobank using females only (based on data field 4079). This GWAS is corrected for the first 10 principal components. The results are shown in **Additional file 1: Tables S6 and S7** for the Mendelian randomization and colocalization analysis respectively. For CACNA1C, CACNA1D, and CACNB3 we do not have any genome-wide significant SNPs, and hence we did not perform the Mendelian randomization analysis.

If we first look at the Mendelian randomization analyses for lowering DBP by any mechanism, genetically-proxied reduction in DBP by 10mmHg through any mechanism was associated with lower risk of pre-eclampsia and eclampsia (OR per 10mmHg reduction in DBP 0.40, 95%CI 0.32-0.49, p=4.78x10^-17^) and birthweight of the first child (beta per 10mmHg reduction in DBP 0.120, 95%CI 0.081 – 0.1592, p=1.90x10^-9^), but was not significantly associated with gestational diabetes (OR 0.98, 95%CI 0.84-1.14, p=0.772).

Genetically-proxied ADRB1 inhibition was associated with lower risk of pre-eclampsia and eclampsia (OR per 10mmHg reduction in DBP 0.037, 95%CI 0.05-0.29, p=0.001) and birthweight of the first child (beta per 10mmHg reduction in DBP -0.489, 95%CI -0.754 - -0.224, p=3.09x10^-4^), but was not significantly associated with gestational diabetes (OR 3.97, 95%CI 0.71-22.39, p=0.118). Colocalization analysis supported a shared causal variant for with pre-eclampsia and eclampsia (posterior probability for H4: 60.15%), and for birthweight of the first child (posterior probability for H4: 64.68%).

For calcium channel blockers, the only previously used gene region with significant SNPs is CACNB2. This means that the results for CACNB2 and CCB are the same. Genetically-proxied reduction in DBP by 10mmHg through CACNB2 is associated with lower risk of pre-eclampsia and eclampsia (OR per 10mmHg reduction in DBP 0.101, 95%CI 0.02-0.47, p=0.004), but was not significantly associated with gestational diabetes (OR 0.40, 95%CI 0.08-1.86, p=0.161) and birthweight of the first child (beta per 10mmHg reduction in DBP -0.015, 95%CI -0.171 - -0.201, p=0.878). Colocalization analyses did not suggest there were any shared causal variants in this gene region.

### **Supplementary analysis to check robustness to colocalization prior**

In this section, we explain how we assert how robust our colocalization analyses are with respect to the joint prior. In the paper by Wallace (2020) the behaviour of both the marginal prior and the joint prior are considered. The work asserts that ‘coloc default values for the prior probabilities of single trait association, p_1_, p_2_, are well supported by data across a range of data types, but that the choice of p12 needs careful thought, and is expected to vary according to the pair of traits being considered.’ Following the suggestion for the joint prior (‘The simulations here suggest that p_12_ = 5×10^−5^ provides a reasonable balance between power and false positive calls, but it is unlikely that any single point distribution on p_12_ captures all prior knowledge’), we reran our coloc analysis using this joint prior of p_12_ = 5×10^−5^. The results are shown in **Additional file 1: Table S8**.

This suggested prior (which is somewhat more favourable for H4) only strengthens our findings, as the posterior probability of H4 for ADRB1 is larger for both pre-eclampsia or eclampsia and birthweight of the first child compared to with our initial prior. For the other gene regions, findings stay relatively similar.

In this manuscript, Wallace also suggests using a joint prior p_12_ = 5×10^−6^ ('The results suggest that the coloc default of p_12_=10^−5^ may be overly liberal, with data simulated under H3 having posterior support for H4, particularly for smaller samples, and that p_12_ = 5 × 10^−6^ may be a more generally robust choice.’), which we also performed (**Additional file 1: Table S8**).

Using this more stringent prior on the joint probability gives us somewhat lower posterior odds of H4. We observe that in this case the H4 posterior probability for ADRB1 in pre-eclampsia or eclampsia is somewhat below our significance level (43%), but still an extremely unlikely scenario of having H3 (posterior probability 0.07%). This suggests that it could be a power issue that we do not find enough evidence for H4 in this case (as the more stringent prior simply dilutes the signal from the data). Still, when we run the analysis with ADRB1 and birthweight of the first child, we find that the posterior for H4 is above 0.5. These analyses suggest that our findings are not very sensitive to the choice of the joint prior.

### **Supplementary analyses to check robustness of the Mendelian randomization method**

Since most Mendelian randomization assumptions cannot be tested, we implement methods more robust to violations of the conventional Mendelian randomization assumptions, including MR-Egger, the weighted median method and MR-PRESSO. First, we briefly describe these methods.

MR-Egger regression introduces an intercept in the weighted regression model to account for directional pleiotropy. A non-zero intercept is an indication of horizontal pleiotropy. Under the Instrument Strength Independent of Direct Effects (InSIDE) assumption (Burgess & Thompson, 2017), which assumes that the magnitude of pleiotropic effects is independent of the size of the SNP-risk factor association, MR-Egger provides robust effect estimates even in the case that all instrumental variables are invalid. The weighted median approach calculates the median of the ratio instrumental variable estimates evaluated using each genetic variant individually and uses the inverse of the variance as the weight. This method can still produce valid estimates if up to 50% of the weighted variants are invalid (Rees et al., 2019). Last, the MR-PRESSO analysis detects outlying SNPs. After removing outliers, it provides consistent casual estimates if the remaining, non-outlying SNPs are valid.

The estimates for these different methods are shown in **Additional file 1: Tables S9-S11**. For ADRB1, CACNA1C, and CACNB3, not enough variants were present to do these robust analyses. We observe that all three robust methods suggest genetically predicted decrease in SBP by any mechanism is still associated with lower risk of pre-eclampsia and eclampsia, and increased birthweight of the first child. For gestational diabetes, no significant association is found. For CACNA1D, no significant associations are observed, but this could be due to a lack of precision. For CACNB3, this target is associated with a reduced risk of pre-eclampsia and eclampsia for all three robust methods, but not associated with gestational diabetes or birthweight of the first child. For all calcium channel blockers targets, there is disagreement amongst the methods about the effect on pre-eclampsia and eclampsia. The weighted median method and MR-PRESSO method suggest this should lower the risk of pre-eclampsia and eclampsia. It could be that the MR-Egger method is somewhat underpowered here, as this method in general tends to have less precision.

### **Strobe MR Checklist**

**STROBE-MR checklist of recommended items to address in reports of Mendelian randomization studies (Skrivankova et al., 2021)**

| **Item No.** | **Section** | **Checklist item** | **Relevant text from manuscript** |
| --- | --- | --- | --- |
| 1 | **TITLE and ABSTRACT** | Indicate Mendelian randomization (MR) as the study’s design in the title and/or the abstract if that is a main purpose of the study | See title (Safety of beta-blocker and calcium channel blocker antihypertensive drugs in pregnancy: a Mendelian randomization study) and Design in Abstract (‘Two-sample Mendelian randomization analysis.’). |
|  | **INTRODUCTION** |  |  |
| 2 | **Background** | Explain the scientific background and rationale for the reported study. What is the exposure? Is a potential causal relationship between exposure and outcome plausible? Justify why MR is a helpful method to address the study question | See Abstract (background/methods section) and first and last paragraph of the Introduction (‘Treatment … growth restriction’, ‘in the … and birthweight’). |
| 3 | **Objectives** | State specific objectives clearly, including pre-specified causal hypotheses (if any). State that MR is a method that, under specific assumptions, intends to estimate causal effects | See Abstract Objectives section (‘We aimed … in pregnancy’) and third paragraph of Introduction (‘In this… and birthweight.’) . See also Methods section under Mendelian randomization (‘MR is … (exclusion restriction)’. |
|  | **METHODS** |  |  |
| 4 | **Study design and data sources** | Present key elements of the study design early in the article. Consider including a table listing sources of data for all phases of the study. For each data source contributing to the analysis, describe the following: |  |
|  | a) | Setting: Describe the study design and the underlying population, if possible. Describe the setting, locations, and relevant dates, including periods of recruitment, exposure, follow-up, and data collection, when available. | Table 1/original studies/methods (data sources). |
|  | b) | Participants: Give the eligibility criteria, and the sources and methods of selection of participants. Report the sample size, and whether any power or sample size  calculations were carried out prior to the main analysis | Table 1/original studies. |
|  | c) | Describe measurement, quality control and selection of genetic variants | See Data sources under Methods section and Instrumental variables under Methods section. |
|  | d) | For each exposure, outcome, and other relevant variables, describe methods of assessment and diagnostic criteria for diseases | Table 1/original studies/Data Sources. |
|  | e) | Provide details of ethics committee approval and participant informed consent, if relevant | See Methods under data sources. |
| 5 | **Assumptions** | Explicitly state the three core IV assumptions for the main analysis (relevance, independence and exclusion restriction) as well assumptions for any additional or sensitivity analysis | See Methods under Mendelian randomization. |
| 6 | **Statistical**  **methods: main analysis** | Describe statistical methods and statistics used |  |
|  | a) | Describe how quantitative variables were handled in the analyses (i.e., scale, units, model) | See Table 1. |
|  | b) | Describe how genetic variants were handled in the analyses and, if applicable, how their weights were selected | See second paragraph of Methods section. |
|  | c) | Describe the MR estimator (e.g. two-stage least squares, Wald ratio) and related  statistics. Detail the included covariates and, in case of two-sample MR, whether the same covariate set was used for adjustment in the two samples | See Data section under Mendelian randomization. |
|  | d) | Explain how missing data were addressed | Not applicable (we only use summary data). |
|  | e) | If applicable, indicate how multiple testing was addressed | See Data section under Mendelian randomization. |
| 7 | **Assessment of**  **assumptions** | Describe any methods or prior knowledge used to assess the assumptions or justify their validity | We use known drug targets for systolic blood pressure. |
| 8 | **Sensitivity analyses and additional analyses** | Describe any sensitivity analyses or additional analyses performed (e.g. comparison of effect estimates from different approaches, independent replication, bias analytic techniques, validation of instruments, simulations) | We perform colocalization analyses for ADRB1 (see Results and Supplementary Note), robust methods (Supplementary Note), different exposure datasets (SBP in females only, DBP); see Discussion/Supplementary Note |
| 9 | **Software and preregistration** |  |  |
|  | a) | Name statistical software and package(s), including version and settings used | See Methods (‘All analyses were performed in R version 4.1.0. ‘ ,  ‘This was implemented in the TwoSampleMR package version 0.5.6’,  ‘For these analyses, the R package coloc (version 5.1.0) was used.’ |
|  | b) | State whether the study protocol and details were pre-registered (as well as when and where) | The analysis plan is described in Figure 1. |
|  | **RESULTS** |  |  |
| 10 | **Descriptive data** |  |  |
|  | a) | Report the numbers of individuals at each stage of included studies and reasons for exclusion. Consider use of a flow diagram | See Figure 1/Table 1/original studies/Supplementary Figure 1. |
|  | b) | Report summary statistics for phenotypic exposure(s), outcome(s), and other relevant variables (e.g. means, SDs, proportions) | See Table 1 and original studies |
|  | c) | If the data sources include meta-analyses of previous studies, provide the assessments of heterogeneity across these studies | This is only the case for our exposure data, see online methods of original study (Evangelou et al, 2018; online methods section ‘Meta-analyses of discovery datasets’). |
|  | d) | For two-sample MR:   1. Provide justification of the similarity of the genetic variant-exposure associations between the exposure and outcome samples 2. Provide information on the number of individuals who overlap between the exposure and outcome studies | 1. All studies use European ancestry individuals (Table 1). 2. There is some overlap between the exposure dataset (SBP) and the outcome dataset for birthweight of the first child (both use UK Biobank). This can be at most 20% overlap. |
| 11 | **Main results** |  |  |
|  | a) | Report the associations between genetic variant and exposure, and between genetic variant and outcome, preferably on an interpretable scale | See supplementary Tables, and original studies. |
|  | b) | Report MR estimates of the relationship between exposure and outcome, and the measures of uncertainty from the MR analysis, on an interpretable scale, such as odds ratio or relative risk per SD difference | We report MR estimates as OR per 10mmHg reduction in SBP/ beta per 10mmHg reduction in SBP. This beta can be interpreted as increase/decrease in categories between low birthweight, average birthweight and high birthweight.  See results section. |
|  | c) | If relevant, consider translating estimates of relative risk into absolute risk for a meaningful time period | Not applicable |
|  | d) | Consider plots to visualize results (e.g. forest plot, scatterplot of associations between genetic variants and outcome versus between genetic variants and exposure) | We provide a forest plot in Figure 2 where we provide the estimated effect of the drug and provide comparison with SBP in general. Also a locus plot is provided in Figure 3. |
| 12 | **Assessment of assumptions** |  |  |
|  | a) | Report the assessment of the validity of the assumptions | We provide F-statistics in Table 2 for instrument strength and the R-squared. |
|  | b) | Report any additional statistics (e.g., assessments of heterogeneity across genetic variants, such as *I^2^*, Q statistic or E-value) | We did do, where possible, robust analyses (Supplementary Note, Supplementary Tables). |
| 13 | **Sensitivity analyses and additional analyses** |  |  |
|  | a) | Report any sensitivity analyses to assess the robustness of the main results to violations of the assumptions | We provide a locus plot for ADRB1 in Figure 3 and did colocalization analysis (Supplementary Tables). We did do, where possible, robust analyses (Supplementary Note, Supplementary Tables). |
|  | b) | Report results from other sensitivity analyses or additional analyses | See 4^th^ paragraph Discussion and Supplementary Note. |
|  | c) | Report any assessment of direction of causal relationship (e.g., bidirectional MR) | Not applicable |
|  | d) | When relevant, report and compare with estimates from non-MR analyses | See Discussion |
|  | e) | Consider additional plots to visualize results (e.g., leave-one-out analyses) | Not applicable |
|  | **DISCUSSION** |  |  |
| 14 | **Key results** | Summarize key results with reference to study objectives | See Discussion. |
| 15 | **Limitations** | Discuss limitations of the study, taking into account the validity of the IV assumptions, other sources of potential bias, and imprecision. Discuss both direction and magnitude of any potential bias and any efforts to address them | See Discussion |
| 16 | **Interpretation** |  |  |
|  | a) | Meaning: Give a cautious overall interpretation of results in the context of their limitations and in comparison with other studies | See Discussion paragraph 1-3. |
|  | b) | Mechanism: Discuss underlying biological mechanisms that could drive a potential causal relationship between the investigated exposure and the outcome, and whether the gene-environment equivalence assumption is reasonable. Use causal language carefully, clarifying that IV estimates may provide causal effects only under certain assumptions | See Discussion paragraph 3. |
|  | c) | Clinical relevance: Discuss whether the results have clinical or public policy relevance, and to what extent they inform effect sizes of possible interventions | See Discussion |
| 17 | **Generalizability** | Discuss the generalizability of the study results (a) to other populations, (b) across other exposure periods/timings, and (c) across other levels of exposure | See Discussion |
|  | **OTHER INFORMATION** |  |  |
| 18 | **Funding** | Describe sources of funding and the role of funders in the present study and, if applicable, sources of funding for the databases and original study or studies on which the present study is based | See Funding section |
| 19 | **Data and data sharing** | Provide the data used to perform all analyses or report where and how the data can be accessed, and reference these sources in the article. Provide the statistical code needed to reproduce the results in the article, or report whether the code is publicly accessible and if so, where | See Availability of data and materials section. |
| 20 | **Conflicts of Interest** | All authors should declare all potential conflicts of interest | See Competing interests. |


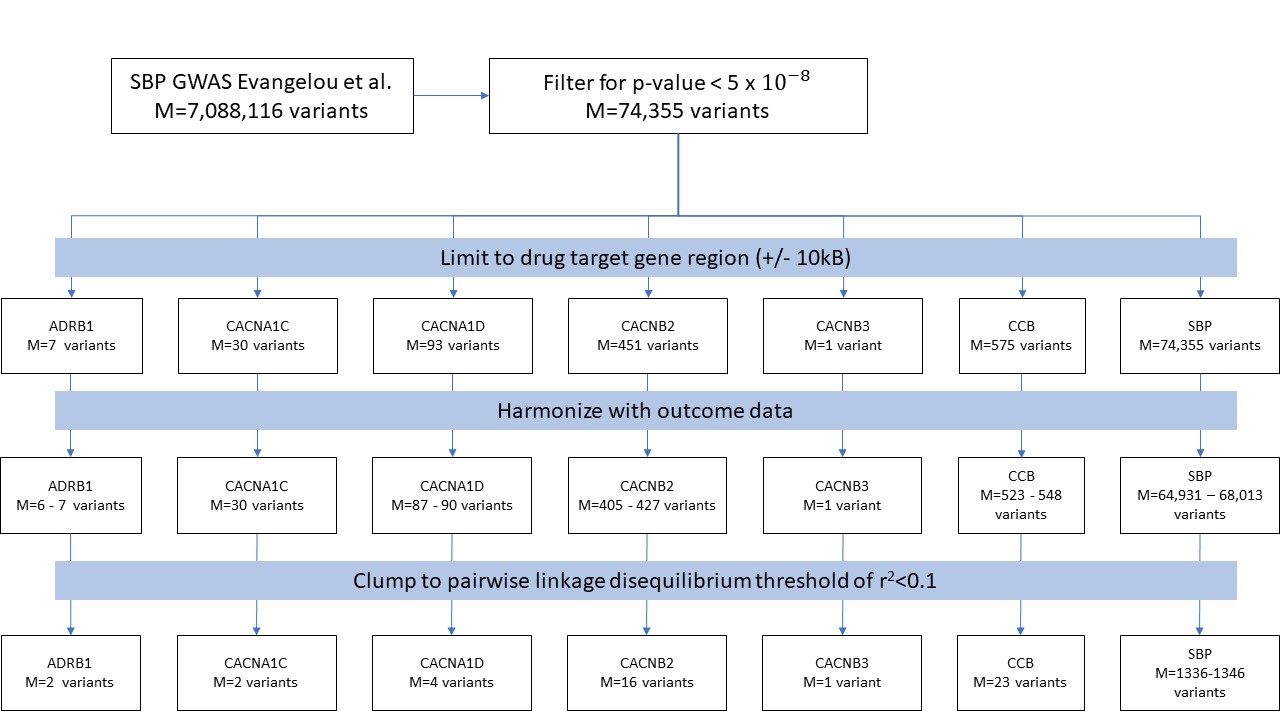


**Figure S1: flowchart detailing instrumental variable selection.**

Abbreviations: SBP = systolic blood pressure, GWAS = genome-wide association study, M= number of markers.

**References**

1. Wallace, C. (2020). Eliciting priors and relaxing the single causal variant assumption in colocalisation analyses. *PLoS genetics*, 16(4), e1008720.
2. Burgess, S., & Thompson, S. G. (2017). Interpreting findings from Mendelian randomization using the MR-Egger method. *European journal of epidemiology*, 32(5), 377-389.
3. Rees, J. M., Wood, A. M., Dudbridge, F., & Burgess, S. (2019). Robust methods in Mendelian randomization via penalization of heterogeneous causal estimates. *PloS one*, 14(9), e0222362.
4. Skrivankova VW, Richmond RC, Woolf BAR, Davies NM, Swanson SA, VanderWeele TJ, et al. Strengthening the Reporting of Observational Studies in Epidemiology using Mendelian Randomisation (STROBE-MR): Explanation and Elaboration. *BMJ*. 2021;375:n2233.
